# The "Domestic" Mosquito

**Published:** 1899-08-05

**Authors:** 


### THE "DOMESTIC" MOSQUITO.

Major Donald Ross, writing as to the possibility of extirpating the mosquito, says that in many places this could be done if people would but take the trouble to set about it. The principal ally of the mosquitos is the stupidity of their victims, for most persons in the tropics, Europeans included, breed their own mosquitos in their own houses, being too lazy or too ignorant to destroy them. Thus they become quite "domestic" animals, or rather pests. He has known people actually refuse to empty out the tubs of putrid water in which mosquito larvæ were pointed out to them, preferring to believe that the insects arose by spontaneous generation from grass or trees. This view saves them trouble, which in the tropics is a great consideration, and gives them an excuse for grumbling at the climate. He says that he has never met anyone in India who has been sufficiently enlightened to keep his own premises free from mosquito larvæ, though this can easily be done.

